# Understanding Circular RNAs in Health, Welfare, and Productive Traits of Cattle, Goats, and Sheep

**DOI:** 10.3390/ani14050733

**Published:** 2024-02-27

**Authors:** Dimitra Kirgiafini, Maria-Anna Kyrgiafini, Theocharis Gournaris, Zissis Mamuris

**Affiliations:** 1 Laboratory of Genetics, Comparative and Evolutionary Biology, Department of Biochemistry and Biotechnology, University of Thessaly, Viopolis, Mezourlo, 41500 Larissa, Greece; 2 Institute of Animal Genetic Improvement, University Center for Research and Innovation PA.K.E.K. “IASON”, University of Thessaly, 38221 Volos, Greece; 3 Averofeio Agri-Food Technological Park of Thessaly, University of Thessaly, Gaiopolis, 41336 Larissa, Greece

**Keywords:** noncoding RNAs (ncRNAs), *Bos taurus*, *Ovis aries*, *Capra hircus*, livestock, biomarkers

## Abstract

**Simple Summary:**

Circular RNAs (circRNAs) are derived from regions that are transcribed but do not code for proteins. Instead, they form covalent closed-loop structures and play a crucial role in many biological processes. This literature review focuses on the function of circRNAs in cattle, goats, and sheep, with a particular emphasis on their impact on economically significant traits such as milk production, meat quality, muscle development, wool production, etc. Additionally, the review explores the potential of circRNAs as biomarkers for the assessment of animal health and welfare. An enhanced understanding of circRNAs’ roles can significantly improve the management of farm animals and the quality of products derived from them. This is increasingly relevant in a context where consumer concerns about animal welfare and sustainable farming practices are growing. The insights provided here pave the way for future studies, highlighting the importance of circRNAs in advancing livestock practices.

**Abstract:**

Circular RNAs (circRNAs) are unique noncoding RNA molecules, notable for their covalent closed-loop structures, which play a crucial role in regulating gene expression across a variety of biological processes. This review comprehensively synthesizes the existing knowledge of circRNAs in three key livestock species: *Bos taurus* (cattle), *Ovis aries* (sheep), and *Capra hircus* (goats). It focuses on their functional importance and emerging potential as biomarkers for disease detection, stress response, and overall physiological health. Specifically, it delves into the expression and functionality of circRNAs in these species, paying special attention to traits critical to livestock productivity such as milk production, meat quality, muscle development, wool production, immune responses, etc. We also address the current challenges faced in circRNA research, including the need for standardized methodologies and broader studies. By providing insights into the molecular mechanisms regulated by circRNAs, this review underscores their scientific and economic relevance in the livestock industry. The potential of circRNAs to improve animal health management and the quality of animal-derived products aligns with growing consumer concerns for animal welfare and sustainability. Thus, this paper aims to guide future research directions while supporting the development of innovative strategies in livestock management and breeding.

## 1. Introduction

The significance of livestock is paramount in the agricultural sector, both economically and as a key source of dietary protein. Livestock value chains are estimated to employ over 1.3 billion people globally, contributing to around 40% of the total agricultural production value [1]. Furthermore, in Europe, animal protein accounts for over 50% of the total protein consumption [2]. Notably, cattle, goats, and sheep are vital within this sector. Specifically, cattle play a significant role in milk and meat production, while goats and sheep, known for their adaptability, are particularly valuable in diverse environments and can thrive on marginal lands unsuitable for plant crops [3]. According to studies, 360 million cattle and 600 million small ruminants contribute 25% of the world’s animal products from such marginal lands [4]. Therefore, these livestock species are not only economically important but also provide essential nutrition and hold cultural value for rural communities.

However, in recent years, the livestock sector has encountered significant challenges and transformations. Climate change has led to extreme weather conditions like high temperatures, floods, and droughts, complicating agricultural production and reducing animal productivity [5]. Concurrently, the growing human population has escalated food demand, necessitating higher productivity [6]. This intensification of production heightens the risk of infectious diseases spreading [7]. Additionally, there is increased consumer concern regarding animal-rearing practices and the ethical procurement of animal-derived food products, underscoring the need for vigilant monitoring of animal welfare [8]. Given these challenges and the push towards more sustainable food systems, it is crucial to understand the molecular mechanisms underlying key physiological processes, such as animal disease resistance, environmental adaptation, etc. Developing biomarkers to monitor animal health and welfare and selecting suitable candidates for breeding programs are pivotal steps in this context, too.

Advancements in high-throughput sequencing, alongside progress in molecular biology and genetics, have brought noncoding RNAs (ncRNAs) into the spotlight. Noncoding RNAs, including microRNAs (miRNAs), long noncoding RNAs (lncRNAs), and circular RNAs (circRNAs), have emerged as key players in the regulation of various cellular processes with broader implications in the regulation of animals’ health, welfare, and production [9,10,11]. Additionally, due to their distinctive properties, many of these molecules have the potential to be used as biomarkers for various disorders and health conditions [8,12,13].

Compared to other ncRNAs, circRNAs remain less studied, with research focusing on their roles in livestock production, health, and welfare being still in its infancy. CircRNAs are distinctive, single-stranded RNA transcripts that form a covalently closed, continuous loop. Typically, precursor mRNAs (pre-mRNAs) are synthesized by RNA polymerase II (Pol II). This process is followed by canonical splicing, as introns are removed and exons are joined to form a linear RNA transcript (mature messenger RNA, mRNA) with a 5′–3′ polarity. However, an alternative splicing mechanism, termed “back-splicing”, can also occur. During back-splicing, the 3′ end of an exon is connected to the 5′ end of the same or an upstream exon, forming a 3′,5′-phosphodiester bond. This results in a unique closed-loop structure with a back-splicing junction site, a hallmark of circRNAs [14,15,16]. Furthermore, circRNAs can be classified into three primary types based on their origin: exon-derived circRNA (ecRNA), intron-derived circRNA (ciRNA), and exon-intron circRNA (EIcircRNA) [17]. Finally, circRNAs are noted for their high cellular stability, evolutionary conservation across species, and resistance to degradation by certain RNA-degrading enzymes [18,19,20].

The objective of this review is to explore and critically assess the roles of circular RNAs (circRNAs) in animal health, welfare, and productivity, especially focusing on their biomarker potential based on the existing literature. As there is an increasing interest in circRNAs for monitoring the welfare of livestock during metabolic, environmental, and management stress, particularly in small ruminants and cattle, in this review, we aim to consolidate current circRNA findings, listing validated circRNAs for evaluating livestock health and welfare. This comprehensive overview seeks to engage the scientific community and facilitate the identification of potential biomarkers, which could be pivotal for future research and applications, particularly considering the multifaceted challenges currently encountered in the livestock industry.

## 2. Materials and Methods

For the present review, we conducted an extensive literature search on circRNAs related to animal health and welfare in July 2023, using PubMed and the Web of Science databases. More specifically, we searched the databases using the term “circular RNA” and all its variants, as follows: #1; “circular RNA” OR “circular RNAs” OR “circRNA” OR “circRNAs” OR “circ RNA” OR “circ RNAs” OR “ciRNA” OR “ciRNAs”. Similarly, for identifying studies associated with animal welfare, we used these terms: #2; “Bos taurus” OR “cow” OR “sheep” OR “Ovis aries” OR “goat” or “Capra hircus” OR “livestock”. The final search formula was a combination of the two terms described above (#1 AND #2). Furthermore, title/abstract fields were applied to our search in PubMed, whereas the topic field was used for the search in Web of Science. After both searches, duplicate articles were removed from subsequent analysis using automation tools (Zotero Citation Manager software, https://www.zotero.org/, accessed on 10 January 2024).

For the screening process that followed, we applied a series of inclusion and exclusion criteria to the identified articles. Specifically, articles were included if they (i) were in the English language, (ii) were in vivo studies on animal subjects of interest (cow, goat, sheep), and/or (iii) were experimental studies reporting differentially expressed (DE) circRNAs. We excluded studies on cell lines (in vitro), reviews, meta-analyses, books, book chapters, conference abstracts, commentaries, replies, and other types of publications with no original data. Articles focusing on reproduction were also excluded, as our focus was on circRNAs related to productive traits potentially serving as biomarkers. Based on the above criteria, the remaining articles were manually screened by title, keywords, and abstract for relevance to our review.

Finally, after collecting the final number of articles, we extracted key information from each one of them for the review. This included the study’s title, authorship details, publication year, country, and journal, as well as the study’s objectives, sample characteristics (e.g., number of animals, biological material used), experimental design, identified differentially expressed circRNAs, validated circRNAs and the implicated genes and pathways affected by DE circRNAs.

## 3. Circular RNAs in Cattle: Shaping Health and Productivity

Cattle (*Bos taurus*) play a pivotal role in global agriculture, functioning as key economic keystones and nutritional reservoirs with far-reaching significance. As integral contributors to the meat and dairy industries, cattle have great economic importance. The mandate to optimize their productivity, health, and welfare converges at the intersection of economic sustainability and scientific progress, particularly in light of the many challenges faced by cattle farming, encompassing disease susceptibility [21], productivity oscillations, and environmental stressors [22,23]. The study of circRNAs can provide a better understanding of the intricate molecular mechanisms governing cattle physiology, presenting avenues for targeted interventions and innovative strategies to address the multifaceted challenges encountered by the cattle farming industry.

Consequently, this section aims to consolidate and synthesize studies elucidating the impact of circRNAs on cattle health and productivity, thus contributing to the scientific discourse and fostering potential advancements in the domain.

### 3.1. Milk Production

Lactation represents a dynamic physiological process essential for milk production, delivering crucial nutrition and immune benefits to the offspring while concurrently meeting the maintenance requirements of the mother [24]. In the context of dairy farming, milk serves as the primary product marketed for human consumption, with cattle contributing to 81% of the total global milk production [25]. Rich in biologically active fatty acids, including saturated fatty acids and conjugated linoleic acid, milk composition not only profoundly affects its flavor and nutritional profile but is also a key consideration in the breeding of dairy cattle, directly influencing the quality of fresh milk [26]. Numerous studies delve into the intricate interplay of circRNAs in milk fat metabolism and the lactation process, providing valuable insights that can serve as a roadmap for enhancing overall milk quality.

One of the pioneering studies in exploring the impact of circRNAs on lactation was conducted by Zhang et al. (2016) [27], who investigated the circRNA profiles in cow mammary glands at 90 and 250 days after giving birth. Notably, 80 circRNAs were identified to originate from all four casein-coding genes (*CSN1S1*, *CSN1S2*, *CSN2*, and *CSN3*). These circRNAs exhibited high expression levels in the mammary tissue of cows after 90 days of lactation, and they were found to contain multiple binding sites for the microRNA miR-2284 family. This suggests the active involvement of circRNAs in modulating the expression of the casein genes during lactation.

Furthermore, in a study led by Chen et al. (2021) [28], mammary gland tissues from three cows were analyzed during dry and peak lactation phases. Through high-throughput sequencing, the research revealed a significant reduction in miR-128 expression attributed to circ11103. Circ11103, highly present in mammary gland tissue, is key in regulating milk fat metabolism by enhancing the production of triglycerides and fatty acids through its interactions with miR-128. This miRNA, in turn, targets and suppresses the *PPARGC1A* gene, which is vital for milk fat metabolism, therefore affecting its function. Consequently, it is hypothesized that the circ11103/miR-128/*PPARGC1A* pathway exerts a regulatory effect on milk fat metabolism and fatty acid synthesis in dairy cows. In a similar approach, Chen et al. (2023) [29] also demonstrated that circRNA-02191 regulates triglyceride and fatty acid components by binding miR-145, therefore mitigating miR-145’s inhibitory effect on *CD36* expression—an essential receptor for the absorption and transport of long-chain unsaturated fatty acids. Consequently, circRNA-02191 modulates the synthesis of unsaturated fatty acids through interactions with miR-145/*CD36*. Collectively, these findings present an innovative approach to enhancing milk quality by investigating the regulatory impact and mechanism of the mentioned pathways. Noteworthy is also the observed impact of both circRNAs (circ11103 and circRNA-02191) on increasing unsaturated fatty acids content while decreasing the relative proportion of saturated fatty acids [28,29].

Similarly, in a study conducted by Liang et al. (2022) [30], the investigation into circRNAs in Holstein cow mammary tissues during early lactation compared to non-lactation was undertaken. Through high-throughput RNA sequencing (RNA-seq), 87 circRNAs were found to be significantly differentially expressed (DE) in peak lactation cows compared to non-lactating ones, with 68 being upregulated and 19 downregulated. These identified circRNAs are speculated to be intricately involved in diverse physiological processes, including fatty acid transport, triglyceride synthesis, as well as inflammation, and immune regulation [30].

Regarding specifically milk fat metabolism, Feng et al. (2022) [31] analyzed circRNA profiles in bovine mammary epithelial cells (BMECs) of cows with a high percentage of milk fat (HMF) and a low percentage of milk fat (LMF) to discern circRNAs associated with milk fat metabolism. The study unveiled a total of 290 DE circRNAs, comprising 142 upregulated and 148 downregulated ones. The host genes of these differentially expressed circRNAs were primarily linked to lipid metabolism, with the most enriched term being cholesterol transport. Among the enriched pathways, the PI3K-Akt signaling pathway took precedence, followed by ECM-receptor interaction and endocytosis, while other involved pathways included mTOR and AMPK. Following the construction of competitive endogenous RNA (ceRNA) networks, the researchers identified four regulatory networks (circ0001122/miR-12043/*LIPG*, circ0007367/miR-331-3p/*CIDEA* and *PML*, and circ0018269/miR-11989/*RORC* and *HPX*), underscoring their pivotal roles in regulating milk lipid metabolism [31]. Similar outcomes were obtained through RNA-seq analysis of mammary epithelial cells (MECs) isolated from cows’ milk with varying milk fat percentages. Transcriptome examination revealed 309 DE circRNAs, with 150 exhibiting upregulation and 159 showing downregulation. The DE circRNAs were predominantly associated with biological processes such as cell migration, locomotion, and localization, and molecular functions like insulin-like growth factor I binding, enzyme binding, and steroid receptor RNA activator RNA binding. Kyoto Encyclopedia of Genes and Genomes (KEGG) pathway analysis highlighted key signaling pathways, including the mTOR signaling pathway, the ECM-receptor interaction, the adherens junction, and the focal adhesion signaling pathway. Finally, the researchers identified five crucial ceRNA networks that potentially regulate mammary gland development, lactation, and milk fat in dairy cows. These networks involve novel circ0011157/bta-miR-1291/*CSF1*, novel circ0011157/bta-miR-1777b/*VDR*, novel circ0011157/bta-miR-1777b/*MECP2*, novel circ0000856/bta-miR-29d-3p/*TET2*, and novel circ0011944/bta-miR-12033/*CD34* [32].

### 3.2. Muscle Growth and Development

Meat constitutes a crucial component of the human diet and has significant implications for both health and the global economy [33]. Cattle meat consumption has demonstrated a consistent and stable trend over time [34]. In the realm of beef production, the intricate relationship between muscle growth in cattle and meat quality is paramount, influencing taste, tenderness, and overall desirability. Factors such as muscle fiber composition, intramuscular fat content, and connective tissue collectively contribute to the sensory attributes that define beef [35]. However, despite the fundamental role of circRNAs in gene regulation and cellular processes, only a limited body of research has explored their specific involvement in muscle growth and development in cattle.

Wei et al. (2017) [36] investigated the role of circRNAs in the muscle development of cattle by comparing embryonic and adult muscle samples from Chinese Qinchuan cattle, which are known for their superior meat quality. Their analysis revealed 828 DE circRNAs. Among these, circLMO7 emerged as one of the most downregulated circRNAs in adult muscle tissue compared to embryonic muscle tissue. The study focused on circLMO7 and demonstrated that, by functioning as a ceRNA for miR-378a-3p, circLMO7 positively regulates the expression of crucial target genes involved in myoblast differentiation and survival. These findings highlight the significant regulatory role of circLMO7 and circRNAs, in general, in the intricate molecular processes that govern myoblast biology.

**Figure 1:**
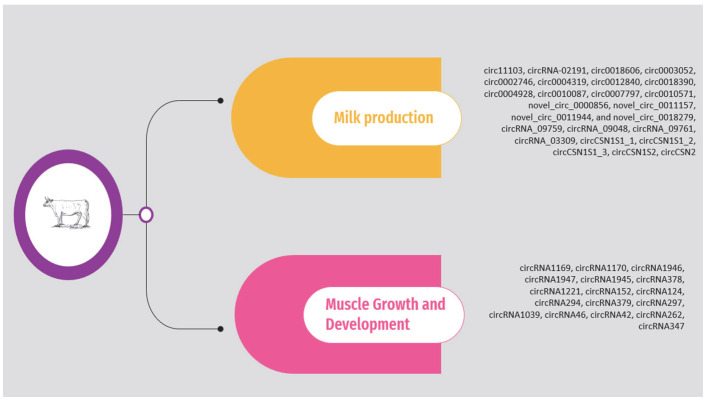
Overview of circRNAs associated with milk production and muscle growth and development in cattle.

The circRNAs associated with milk production and muscle growth and development according to the above studies are presented in Supplementary Table S1 and Figure 1. It should be noted that the list is limited to circRNAs validated through molecular approaches, such as quantitative polymerase chain reaction (qPCR).

### 3.3. Immunity

Immunity in cattle is a pivotal factor with profound implications for both animal health and the overall productivity of the livestock industry. A well-balanced and responsive immune system is critical, playing a key role not only in preventing and combatting infections but also in supporting overall health and performance [37]. Furthermore, there is a growing need to study immunity, particularly since the intensification of milk and beef production inevitably increases the risk of infectious disease spread and exacerbation [38]. Despite the acknowledged significance of immunity in cattle, the exploration of circRNAs in the context of bovine immunity is still in its early stages. The only available study associated with circRNAs and immunity in cattle focused on mastitis, a substantial economic burden and a significant challenge for the dairy industry.

More specifically, in the study by Bai et al. (2022) [39], the expression of circRNAs in *Staphylococcus aureus*-induced mastitis was explored using mammary tissue samples from healthy (HCN) and affected Holstein cows (HCU). Employing RNA-seq, 19 DE circRNAs were identified, with 6 upregulated and 13 downregulated in HCU compared to HCN. Notably, three circRNAs—circRNA2860, circRNA5323, and circRNA4027—showed consistent differential expression in both RNA-seq and qPCR analyses, suggesting their potential as key regulators in mastitis. Importantly, the host genes of these circRNAs, including *TRPS1*, *SLC12A2*, and *MYH11*, may play a direct or indirect role in the development of cow mastitis. Furthermore, DE circRNAs were significantly enriched in RNA polymerase transcription factor binding and the tight junction pathway, processes, and pathways particularly relevant to cow mammary epithelial tissue.

### 3.4. Heat Stress

In recent years, the impacts of global warming have become increasingly evident across various sectors, including the dairy industry. Heat stress (HS) significantly affects dairy cows, resulting in substantial economic losses for the industry annually. HS induces a range of physiological responses and ailments across diverse bodily systems when bovine species experience temperatures exceeding their intrinsic thermoregulatory thresholds. The subsequent chain of consequences includes a noticeable decline in reproductive efficiency, accompanied by a sharp deterioration in overall physiological well-being. This encompasses the onset of diseases, a decrease in reproductive capabilities, a reduction in milk production, and a concurrent decline in the qualitative attributes of the produced milk [40,41,42]. Therefore, recognizing the imperative to address the adverse impacts of heat stress, numerous studies are exploring the involvement of circRNAs in cattle subjected to heat stress.

Zhang et al. (2023) [43] conducted a comprehensive investigation into the impact of heat stress on key biological processes and the potential involvement of circRNAs in the heat stress response. Their study focused on discerning circRNA expression profiles in peripheral blood samples of cows during heat stress, revealing significant alterations. Specifically, the DE circRNAs were found to be associated with five primary signaling pathways: choline metabolism, the PI3K/AKT signaling pathway, the HIF-1 signaling pathway, the longevity-regulating pathway, and autophagy. Moreover, the study pinpointed three specific circRNAs (ciRNA1282, circRNA4205, circRNA12923) as crucial components in the heat stress response. These circRNAs were notable for their enrichment in multiple pathways and their identification as binding to multiple miRNAs.

Another compelling study, conducted by Zeng et al. (2023) [44], delved into the association between circRNAs and hormonal changes within the hypothalamic-pituitary-mammary gland (HPM) axis. The comprehensive analysis spanned various tissues, including blood samples, as well as the hypothalamus, pituitary, and mammary gland tissues. The investigation uncovered distinctive expression profiles of circRNAs in these tissues, with 1680, 1112, and 521 DE circRNAs identified in the hypothalamus, pituitary, and mammary gland, respectively. Significantly, a notable proportion of these circRNAs exhibited upregulation, with 1111, 2461, and 250 circRNAs displaying increased expression, while 569, 1112, and 271 showed downregulation in their respective tissues. Notably, the study pinpointed a subset of 33 circRNAs shared among the hypothalamus, pituitary, and mammary gland, suggesting potential cross-tissue regulatory roles and offering insights into the complex ceRNA networks within the HPM axis. Among the genes targeted by these DE circRNAs were *HSPH1*, *GHRHR*, *PRL*, *GH1*, and *IGF1*. Consequently, the authors propose that dairy cows may adapt to thermal conditions by modulating the expression of various RNAs and pivotal pathways within the HPM axis, influencing hormone secretion and, in turn, impacting lactation performance.

A few studies also focus specifically on the adverse effects of HS on milk production, milk composition, and the health of the mammary gland. Wang et al. (2020) [45] investigated the impact of circRNAs on lactation, as well as their influence on milk quality and composition under conditions of HS. The findings revealed that HS led to a decline in milk quality, accompanied by reduced concentrations of unsaturated fatty acids. Additionally, the study observed alterations in the expression profile of circRNAs in mammary gland tissue under heat stress conditions. Specifically, 38 ciRNAs were identified as DE between heat-stressed cows and the control group. A total of 19 circRNA candidates exhibited upregulation, while 19 showed downregulation in response to heat stress. The researchers also identified four ciRNA-miRNA-mRNA networks with significant implications for lactation under heat stress. These networks included circFCHSD2-miR6516-*CD36*, circHNRNPLL-miR11986Bcd36, circKANSL1-miR345 and miR502b, and miR6516-*CD36*, circMAP7-miR11986b and miR345-*CD36* [45]. Interestingly, these networks featured the critical involvement of *CD36*, a factor previously highlighted in studies exploring circRNAs and milk fat metabolism [29]. Similarly, Qi et al. (2022) [46] aimed to elucidate the unique expression profiles of circRNAs in mammary gland tissues of cattle experiencing HS compared to those raised under normal climatic conditions. Using the advanced technique of RNA-seq, this investigation revealed a set of 95 DE circRNAs, with 80 showing upregulation and 15 demonstrating downregulation. These DE circRNAs may play pivotal roles in regulating lactation through the PRL and IGF signaling pathways by acting as miRNA sponges. Moreover, examination of the host genes of the DE circRNAs revealed a robust association with the regulation of energy metabolism, with the AMPK signaling pathway emerging as the predominant enriched pathway.

**Figure 2:**
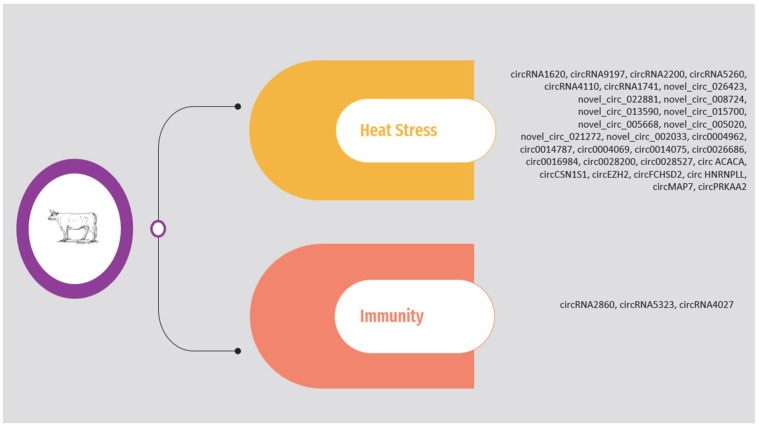
Overview of circRNAs associated with heat stress and immunity in cattle.

As previously mentioned, the circRNAs associated with heat stress and immunity in the above studies are presented in Supplementary Table S1 and Figure 2. The list is limited to circRNAs validated through molecular approaches.

## 4. Circular RNAs in Sheep: Shaping Health and Productivity

Sheep (*Ovis aries*) are invaluable in agricultural systems globally, providing wool, meat, and dairy products. They are central to the textile, culinary, and dairy industries, highlighting their economic and cultural significance [47,48]. Furthermore, their genetic diversity and adaptability to various environments make them vital to both traditional and modern farming practices [49,50].

Several studies of circRNAs offer insights into the complex molecular mechanisms of sheep physiology and their productive traits. Therefore, this section aims to synthesize and analyze research on the role of circRNAs in sheep health, welfare, and productivity, contributing to the scientific discourse and opening pathways for innovations in this crucial area.

### 4.1. Milk Production

Lactation in sheep is a complex physiological process that is essential for milk production, a key component of sheep farming. This process is particularly crucial in areas where dairy cattle are less common, offering an important agricultural practice. Notably, sheep milk is richer in solids compared to cow or goat milk, enhancing its suitability for cheese production [51,52]. Factors such as breed, nutrition, and management practices significantly influence the yield and composition of sheep milk [53]. Similarly, genetics, nutrition, and environmental conditions play a pivotal role in lactation [54]. Recent studies have also shed light on the regulatory role of circRNAs in milk production and lactation, providing valuable insights that could enhance milk quality and improve dairy production in sheep.

In one study, Hao et al. (2020) [55] employed RNA-seq to scrutinize the presence and characteristics of circRNAs in the mammary glands of two distinct sheep breeds with diverse milk production profiles: the Gansu Alpine Merino (GAM) sheep (low-lactating) and the Small-tailed Han (STH) sheep (high-lactating). Notably, 3133 circRNAs (63.9%) were found to be co-expressed in the mammary glands of both STH and GAM sheep, and some of them originated from casein and whey protein-coding genes. Additionally, 33 DE circRNAs were detected between the mammary gland (MG) samples of GAM and STH sheep, including 18 upregulated and 15 downregulated. Noteworthy exclusivity in expression was observed, with five circRNAs exclusively expressed in STH sheep’s MG (circ-014121, circ-007220, circ-19403, circ-015111, and circ-004632) and four circRNAs exclusively expressed in GAM sheep’s MG (circ-004110, circ-021253, circ-017116, and circ-003297). Most parental genes of DE circRNAs were enriched in heterocyclic compound binding, catalytic, and kinase activity. Finally, the researchers constructed a miRNA-circRNA-mRNA network, predicting a total of 18 target miRNAs and 25 mRNAs associated with eight circRNAs. Interestingly, some of these miRNAs have been previously linked to bovine mammary gland development and lactation [56,57,58].

The same team of researchers continued its study on circRNAs and milk production in sheep. In a subsequent study, Wu et al. (2022) [59] explored the role of circ_015343 in the mammary gland tissue of the same sheep breeds: Small Tail Han sheep and Gansu Alpine Merino sheep. They found that circ_015343, originating from the aminoadipic semialdehyde synthase (*AASS*) gene, had a high expression level in the mammary gland during peak lactation and varied expression in different tissues, being least in the *longissimus dorsi* muscle. The study also revealed that inhibiting circ_015343 (using si-circ_015343) increased the viability and proliferation of mammary epithelial cells. Inhibition of circ_015343 influenced the expression of key genes related to milk fat synthesis (*ACACA*, *FABP4*, *SREBP1*) and cell proliferation (*CDK2*, *CDK4*, *PCNA*) while reducing the expression of its parent gene, *AASS*. Notably, sheep breeds with higher milk yield and fat content showed lower levels of circ_015343 expression. Finally, the study highlighted a circRNA–miRNA–mRNA interaction network, suggesting that circ_015343 binds certain microRNAs to regulate genes involved in mammary gland development and lactation. These findings indicate that circ_015343 plays a significant role in regulating lactation performance, potentially contributing to the observed differences in milk yield and composition between the two sheep breeds.

Using a different approach, Wang et al. (2021) [60] conducted a study to investigate the expression profile of circRNAs in mammary glands from the same ewes during peak lactation and the non-lactation period. They identified 3278 circRNAs in lactating mammary gland tissues and 1756 circRNAs in non-lactating mammary gland tissues. Interestingly, 1108 circRNAs were found to be expressed in both lactation and non-lactation periods. Furthermore, the researchers discovered that in ovine mammary gland tissue during peak lactation, circular RNAs were produced by four casein-coding genes (*CSN2*, *CSN1S1*, *CSN1S2*, and *CSN3*), as well as two whey protein-coding genes (*BLG* and *LALBA*). They also identified 41 DE circRNAs. Among these, 40 circRNAs were upregulated, while one was downregulated in mammary gland tissue during peak lactation. To gain further insights, the researchers conducted a GO enrichment analysis of the parental genes associated with the DE circRNAs. The analysis revealed that these genes were associated with various terms such as biological regulation, membrane-bounded organelle, protein binding, ATP binding, and ion binding being particularly enriched. Lastly, the study identified a total of 1423 target microRNAs that interacted with the 41 DE circRNAs. Some of these microRNAs were linked to mammary gland development and lactation.

Similarly, Chen et al. (2022) [61] investigated ncRNA profiles in Hu ewes’ mammary glands during different lactation periods (perinatal, 6 days postpartum, and 25 days postpartum) to understand their roles in mammary gland development and lactation. They identified several DE circRNAs: 348 in early lactation vs. perinatal, 373 in peak lactation vs. perinatal, and 36 in peak lactation vs. early lactation. Between them, novel_circ_0010160 was DE in all comparisons. The most highly expressed circRNAs were also novel_circ_0011066, novel_circ_0011021, and novel_circ_0010252, linked to genes *SLTM*, *USP3*, and *SLC39A8*, respectively, which are crucial for mammary stem cell differentiation, cell cycle regulation, and mammary gland expansion. Furthermore, the study found that parental genes of DE circRNAs were significantly enriched in various GO terms and KEGG pathways, with notable differences in enrichment across lactation stages. Specifically, significant GO terms for early lactation vs. perinatal included mitotic cell cycle organization and chromatin binding, with KEGG enrichment in ECM-receptor interaction and adipocytokine signaling. For peak lactation vs. perinatal, the focus was on cell cycle organization and membrane-bounded organelles, and similarly, in peak lactation vs. early lactation, organelle organization and cytosol were notable GO terms. Finally, the study also pinpointed key circRNA biomarkers (novel_circ_0010460, novel_circ_0004804, and novel_circ_0006544) and constructed a ceRNA network featuring novel_circ_0006589, oar_miR_432, and *PRADC1*, highlighting their potential roles in mammary gland development and lactation processes.

### 4.2. Muscle Growth and Development and Meat Quality

The relationship between muscle growth, intramuscular fat, and meat quality in sheep is complex and interconnected. Specifically, the quality of sheep meat is known to vary at different growth stages due to changes in muscle physiology and biochemistry [62]. Intramuscular fat also plays a crucial role in determining meat quality, influencing attributes such as tenderness, juiciness, and flavor [63]. However, it has been proven that in addition to external factors like management and environmental conditions, intrinsic factors related to the animal itself, particularly genetic characteristics, significantly affect meat quality traits [64]. Recent studies have also begun to explore the role of circRNAs in the complex interaction between muscle growth, intramuscular fat, and meat quality in sheep, as a substantial number of studies exist.

In their study, Li et al. (2017) [65] explored the role of circRNAs in sheep muscle growth and development, a relatively uncharted area at the time. They analyzed muscle samples from adult female Kazakh sheep and embryos, identifying 5086 DE circRNAs. This comparison revealed significant changes in circRNA expression patterns between embryonic and adult muscle tissues. Furthermore, the researchers conducted GO and KEGG pathway enrichment analyses on the hosting genes of these circRNAs, discovering that many are involved in key biological processes related to muscle growth and development. Notably, pathways like the AMPK signaling pathway, ECM-receptor interactions, ErbB signaling pathway, and the mTOR signaling pathway were implicated. The study’s groundbreaking contribution was the construction of a circRNA–miRNA–mRNA network, demonstrating the complex interactions among these molecules in muscle development. They specifically highlighted circRNAs like circRNA 0000385, circRNA 0000582, and circRNA 0001099, which have multiple target sites for muscle-related miRNAs, such as miR-143, miR-133, and miR-23.

One of the pioneering studies in this field was also conducted by Cao et al. (2018) [66] with the primary objective of identifying and annotating circRNAs in the *longissimus dorsi* muscle of sheep. Specifically, the researchers chose Kazakh sheep for their study, given its classification as a meat-type breed characterized by a relatively slow growth rate and low meat yield. Thus, enhancing the meat characteristics of Kazakh sheep has become a central focus in stockbreeding. A total of 886 circRNAs originating from 729 genes were identified within the skeletal muscle of sheep through RNA-seq. Then, the researchers explored the interactions between circRNAs and microRNAs, discovering numerous circRNAs that interacted with muscle-specific miRNAs integral to muscle growth and development. Notably, one circRNA, circ776, exhibited particularly robust interactions with these miRNAs. Finally, to probe the functional roles of circRNAs, the team conducted an enrichment analysis of genes associated with circRNAs. This analysis revealed several pivotal signaling pathways, including the transforming growth factor-beta (TGF-beta) signaling pathway, mammalian target of rapamycin (mTOR) signaling pathway, Wnt signaling pathway, and MAPK signaling pathway, all recognized for their significant contributions to muscle development and growth.

Another study by Bao et al. (2022) [67] focused on exploring the circRNA–miRNA–mRNA regulatory networks impacting the growth and development of the *longissimus thoracis* (LT) muscle in Tibetan sheep. The research primarily identified significant variations in meat quality at four distinct growth stages (4 months, 1.5 years, 3.5 years, and 6 years), observing a decline in meat tenderness as the sheep aged. Using RNA-seq, the team discovered 11,749 circRNAs, with 3851 being co-expressed across all age groups, while DE circRNAs between the four groups were also identified. Notably, certain circRNAs, such as circ_000631, circ_000281, and circ_003400, were found to interact with miRNAs like miR-29-3p and miR-185-5p. This interaction influences the expression of genes, including *LEP*, *SCD*, and *FASN,* within the AMPK signaling pathway. The study elucidated that this network plays a role in the muscle fiber transition from oxidative to glycolytic types, which is associated with a decrease in intramuscular fat and an increase in muscle fiber diameter, factors contributing to enhanced meat tenderness. Additionally, correlation analyses revealed that specific circRNAs significantly correlate with slaughter performance and meat quality traits such as shear force, intramuscular fat content, and crude protein content. The findings indicate a strong association between circRNAs and key indicators of meat quality in the LT muscle growth of Tibetan sheep. Finally, the study suggests that slaughtering Tibetan sheep at 1.5 years optimizes meat quality due to specific muscle growth characteristics.

Similarly, Liu et al. (2022) [68] conducted a comprehensive study to investigate the expression profile and functions of circRNAs in skeletal muscle using RNA-seq. Their study focused on *longissimus dorsi* samples obtained from three different age groups of Duolang sheep: 90-day-old fetuses (F90), 30-day-old lambs (L30), and three-year-old adult sheep (A3Y). Analyses revealed similar circRNA expression patterns in the L30 and A3Y groups, which differed significantly from the F90 group. Notably, the L30 vs. A3Y comparison exhibited the fewest DE circRNAs, while F90 vs. A3Y showed the most significant differences, highlighting substantial transcriptional changes between prenatal and postnatal muscle development. During muscle development, upregulated DE circRNAs were primarily associated with tissue development and energy metabolism. Specifically, in the L30 vs. F90 comparison, upregulated DE circRNAs were enriched in pathways related to muscle constituents and protein homodimerization activity. In the L30 vs. A3Y group, upregulated DE circRNAs were linked to epithelial cell development and transcription coregulator activity, while downregulated circRNAs affected cell cycle pathways. Importantly, KEGG pathway analysis revealed significant enrichment in the AMPK and PI3K-Akt signaling pathways for the L30 vs. A3Y group, metabolic and myocardial-related pathways for the L30 vs. F90 group, and a unique set of pathways, including TGF-beta, PI3K-Akt, AMPK, and FoxO signaling for the A3Y vs. F90 group. Additionally, the study constructed a circRNA–miRNA network and observed that several circRNAs contained at least two conserved target sites for miRNAs associated with muscle development. For example, circ-023984 was found to have binding sites for miR-133 and miR-125, both recognized as regulators of muscle growth and development. Finally, it was suggested that circCHRNG functions as a miRNA sponge (miR-133), influencing the levels of *SRF* and *MEF2A*, which in turn govern the proliferation of skeletal muscle satellite cells.

In another study, Cui et al. (2022) [69] investigated muscle fiber formation in Tan and Dorper sheep by comparing their transcriptomes. The study involved analyzing differentially expressed genes (DEGs) and ncRNAs in three Tan and three Dorper sheep, focusing on the *longissimus dorsi* and *biceps femoris* muscles. The *longissimus dorsi* and the *biceps femori* of Tan sheep and Dorper sheep displayed significantly differential expression levels. More specifically, 91 DE circRNAs were identified in the *longissimus dorsi*, whereas 95 DE circRNAs were identified in the *biceps femori* of the two sheep. Subsequently, GO and KEGG analyses were also conducted. The GO analysis showed that the parental genes of DE circular RNAs were mainly enriched in myofibril and glycogen metabolic pathways, and the KEGG analysis revealed significant enrichment in pathways like Ca^2+^ signaling, FoxO signaling, and AMPK signaling. Then, the ceRNA network that was constructed highlighted important subnetworks, such as circ_0017336 with targets *FBXL5*, *ACACB*, and *EXOC6*, and LNC_014172 and LNC_003716 with their targets, all interacting through miR-23a response elements. This suggests a significant role of ncRNAs in muscle fiber formation. The study also detailed specific ceRNA networks for *longissimus dorsi* and *biceps femoris* tissues, illustrating complex interactions among various DE lncRNAs, miRNAs, circRNAs, and mRNAs. Finally, the study explored the function of circ_0017336 as a ceRNA in sheep myoblasts. By transfecting sheep myoblasts with the interfering fragment si-circ_0017336, they observed a significant increase in miR-23a expression and a decrease in *FBXL5* expression in skeletal muscle cells, indicating its potential role in muscle fiber development.

A quite different study was performed by Zhao et al. (2021) [70] aimed to investigate the expression of circRNAs in the intramuscular fat (IMF) of Aohan fine-wool sheep (AFWS) at different ages and explore their potential regulatory roles in IMF deposition. The researchers collected samples of *longissimus dorsi* muscle from 2-month-old and 12-month-old sheep and measured the IMF content. Subsequently, they performed RNA-seq to identify circRNAs and analyzed their expression patterns. The study revealed that the IMF content in 12-month-old sheep was significantly higher than that in 2-month-old sheep. Regarding RNA-seq, a total of 104 DE circRNAs were identified between the two age groups. The researchers also conducted functional enrichment analysis, which indicated that the enriched pathways included lipid transport, negative regulation of the canonical Wnt signaling pathway, fat digestion and absorption, and sphingolipid metabolism. These pathways are associated with various biological processes, such as lipid metabolism and muscle development. Furthermore, the research team constructed a regulatory network based on the potential interactions between DE circRNAs and microRNAs. Among these interactions, they identified a circRNA-microRNA pair, circRNA4557-miR-149-5p, which may have a targeting relationship and could play a role in the molecular mechanism of sheep IMF deposition. Finally, the researchers also suggest that circRNA2800, circRNA2441, circRNA328, and ciRNA67 may have regulatory roles in IMF deposition in AFWS.

### 4.3. Wool

Wool, a natural fiber obtained from sheep, is renowned for its versatility, durability, and excellent insulating properties. Sheep farming plays a crucial role in wool production, with practices varying globally based on climate, breed, and purpose [71]. The growth of wool is a complex physiological and biochemical process as it is intricately influenced by genetics, environmental conditions, such as climate and pasture quality, and the nutrition of the sheep. Central to this process is the development of hair follicles (HF). The yield and quality of wool are directly linked to the health and development of these follicles [72,73]. Recent scientific advancements have shed light on the role of ncRNAs in wool growth, indicating that they act as important post-transcriptional regulators of gene expression during hair follicle development [74]. Some studies presented below have explored the role of circRNAs on wool production and wool follicle development in sheep.

Zhao et al. (2020) [74] delved into the regulatory functions of circRNAs in the growth of fine wool in Aohan fine-wool sheep (AFWS). Utilizing RNA-seq analysis on samples from sheep shoulder skin at embryonic day 90, embryonic day 120, and birth, the researchers identified a total of 8753 circRNAs, among which 918 demonstrated differential expression across the three developmental stages. Through GO and KEGG analyses, the study unveiled that the source genes of these circRNAs were predominantly linked to cellular component organization, regulation of primary metabolic processes, tight junctions, as well as the cGMP-PKG and AMPK signaling pathways. The researchers also proposed the potential pivotal roles of specific circRNAs, such as circ_0005720, circ_0001754, circ_0008036, circ_0004032, circ_0005174, circ_0005519, and circ_0007826, in the regulation of wool follicle growth in AFWS.

Similarly, in the study conducted by Lv et al. (2020) [75], an extensive investigation was carried out to explore the expression patterns of circRNAs within the hair follicles of Hu sheep lambskin. Specifically, the researchers compared circRNA expression profiles between two groups, small waves (SM) and straight wool (ST) sheep, using RNA-seq. Their findings revealed the presence of 114 DE circRNAs originating from various genes. These circRNAs exhibited distinct expression patterns corresponding to different stages of hair follicle development. Functional enrichment analysis of the parental genes associated with these circRNAs highlighted their involvement in critical biological processes related to skin development, hair growth, and keratinocyte proliferation. Notably, the TGF-beta pathway and Notch pathway also emerged as highly enriched pathways. Furthermore, the study proposed an intriguing regulatory role for circRNAs as ceRNAs, orchestrating interactions with miRNAs and protein-coding genes (PCGs) within a complex network. Particularly noteworthy was the identification of 129 miRNAs that could potentially bind to the 114 DE circRNAs, with some well-known miRNAs, such as miR-10a, miR-125b, miR-143, miR-let-7a, miR-199a-3p, miR-200a, and miR-200b, which are known to have a substantial influence on hair follicle growth and development.

Finally, a more comprehensive approach was employed by Zhao et al. (2021) [76] to gain deeper insights into the genetic and biological underpinnings of hair follicle development and wool-related traits in Merino sheep. Specifically, they utilized transcriptome analysis, methylome analysis, and genome-wide association studies (GWAS) to investigate the molecular signatures and candidate genes associated with wool traits and growth in sheep. The study revealed a total of 41,369 circRNAs originating from 6477 parental genes. These circRNAs exhibited stage-specific expression patterns throughout hair follicle morphogenesis, and their expression levels were organized into six clusters based on their developmental trends. To gain a better understanding of the roles of these circRNAs, GO and KEGG analyses were conducted on their parental genes. The results showed significant enrichment in various biological processes related to skin development, neurogenesis, and internal organogenesis. Moreover, ceRNA network analysis uncovered interactions between circRNAs, miRNAs, and protein-coding genes (PCGs). Several circRNAs were found to regulate the expression of PCGs by acting as miRNA sponges. The ceRNA network was notably enriched in biological processes such as skin development, keratinocyte proliferation, and epidermal development. Based on these findings, the study proposed five circRNAs (circRNA.18823, circRNA.23725, circRNA.29701, circRNA.41342, circRNA.688) that could function as ceRNAs to modulate the expression of stage-specific PCGs during hair follicle morphogenesis. For example, circRNA.18823 and circRNA.688 were identified as potential regulators of *FGF7* expression, a gene pivotal in skin development and cell proliferation.

**Figure 3:**
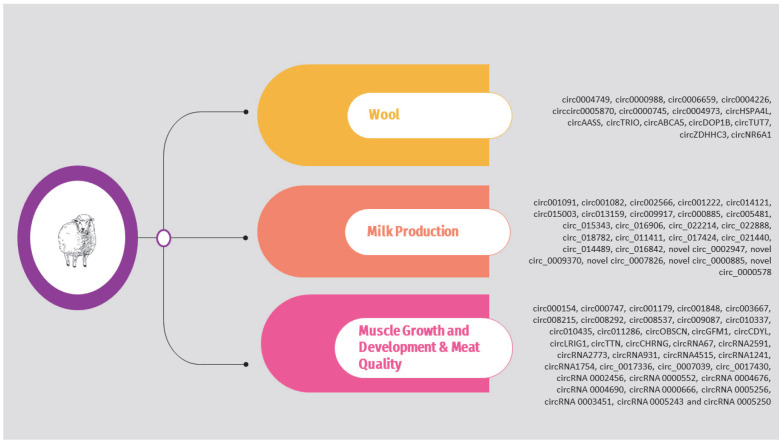
Overview of circRNAs associated with wool, milk production, and muscle growth and development in sheep.

Regarding sheep, the circRNAs identified in the above studies are presented in Supplementary Table S2 and Figure 3. It should be noted that, as previously, the list is limited to circRNAs validated through molecular approaches such as qPCR.

### 4.4. Immunity

Immunity plays a critical role in animal health and significantly affects the productivity of the livestock industry. Despite this importance, studies of circRNA in sheep have primarily concentrated on diarrhea, a prevalent disease in young farm animals, predominantly caused by *Escherichia coli* (*E. coli*) F17.

Jin et al. (2018) [77] conducted a study investigating the expression profiles of circRNAs in the spleens of lambs exhibiting resistance or susceptibility to *Escherichia coli* F17-induced diarrhea. The primary objective was to comprehend the involvement of circRNAs in the immune response and resistance to bacterial infections. After collecting spleen samples from lambs that were either resistant or susceptible to *E. coli* F17-induced diarrhea, RNA-seq was performed. A total of 60 DE circRNAs were identified, with 31 upregulated and 29 downregulated in the resistant group compared to the susceptible group. Furthermore, the researchers employed bioinformatics tools to predict the potential functions of these DE circRNAs, revealing their potential involvement in processes associated with pili adhesion to the intestinal mucosa, a critical factor in bacterial colonization and infection. However, the precise roles of these circRNAs in disease resistance remain largely unknown. Notably, despite the identification of enriched pathways, such as the estrogen signaling pathway, protein processing in the endoplasmic reticulum, and regulation of the actin cytoskeleton, the specific contributions of these pathways to disease resistance remain elusive.

Similarly, Chen et al. (2022) [78] conducted RNA-seq to analyze circRNAs and miRNAs in the jejunum of lambs with varying responses to *E. coli* F17. They identified 16,534 circRNAs and 271 miRNAs, including both novel and previously annotated types. Among these, they found 214 DE circRNAs between *E. coli* F17-antagonism (AN) and F17-sensitive (SE) lambs, with 90 being upregulated and 124 downregulated. The source genes of these DE circRNAs were mainly involved in metabolic pathways and intestinal inflammation pathways. Additionally, 44 circRNAs were identified as potential biomarkers for *E. coli* infection, with novel_circ_0000180, novel_circ_0000365, and novel_circ_0000027 being particularly noteworthy. The research also established circRNA-related ceRNA networks featuring 46 circRNA–miRNA–mRNA competing triplets.

### 4.5. Environmental Stress

Environmental stressors, such as climatic changes and resource scarcity, significantly impact the physiological and behavioral responses of sheep. Yet, research in this area is limited, with only a single study addressing the role of circRNAs in sheep under environmental stress. Specifically, Guo et al. (2022) [79] conducted a compelling study investigating the coevolution of circRNAs in the rumen epithelium of Tibetan sheep in response to cold-season nutritional stress in conjunction with their microbiota and metabolites. The research involved the categorization of sheep into warm and cold-season groups, followed by high-throughput sequencing and other techniques to analyze circRNAs, microbiota, and metabolites. Notably, the study revealed significant alterations in the composition and abundance of rumen epithelial circRNAs under cold-season nutritional stress. Specifically, 56 DE circRNAs were identified, with 29 upregulated and 27 downregulated during the cold season. Analysis of the source genes of DE circRNAs indicated improvements in the energy metabolism and immune defense of Tibetan sheep in response to cold-season stress. Furthermore, an impact on the nutritional stress of Tibetan sheep during the cold season was also indicated by the miRNAs and genes targeted by the circRNAs identified. Finally, the study unveiled a correlation between changes in circRNA expression and alterations in the rumen microbiota and metabolites, implying a coevolutionary relationship between circRNAs and the rumen ecosystem.

### 4.6. Metabolism

Metabolism also plays a fundamental role in the health and productivity of sheep, influencing their growth, reproduction, and overall well-being. Zhang et al. (2022) [80] conducted a study to explore the involvement of circRNAs in the feed efficiency of Hu lambs by performing circular RNA-seq in the liver of Hu sheep with High-RFI (High residual feed intake) and Low-RFI (Low residual feed intake). The researchers identified 219 DE circRNAs. Notably, these circRNAs predominantly originated from genes associated with immunity response and metabolism-related pathways, suggesting a potential regulatory role in these processes concerning feed efficiency. Examination of the ceRNA regulatory networks further indicated that circRNA-targeted genes were linked to both metabolism and immunity. Furthermore, the authors pinpointed a significant single nucleotide polymorphism (SNP) in the target gene *RTP4*, strongly associated with RFI, a key indicator of feed efficiency. This discovery implies that circRNAs may actively participate in the regulation of feed efficiency through their interactions with target genes. The identification of an RFI-associated SNP in the target gene *RTP4* serves as a foundation for future research into the intricate regulatory mechanisms of circRNAs in the context of feed efficiency.

In a different study, He et al. (2022) [81] analyzed the miRNA and circRNA profiles in the tail fat tissue of Sunite sheep, a distinct breed known for their hardiness and adaptability to harsh environments, focusing on their roles in fat metabolism. The researchers collected adipose tissue samples from the tail fat of Sunite rams at 6, 18, and 30 months of age and performed RNA-seq. The study’s key findings include the identification of 1942 miRNAs, of which 392 were novel, and 17,531 circRNAs in the deposited fat tissue of Sunite sheep. Notably, *UTRN* and *ACACA* emerged as the most common host genes for these circRNAs. *UTRN* is associated with pig intramuscular fat, while *ACACA* is crucial in fatty acid synthesis. Furthermore, they identified 93 DE circRNAs in the comparison between 30-month and 6-month-old sheep, 89 DE circRNAs between 30-month and 18-month-old sheep, and 66 DE circRNAs between 18-month and 6-month-old sheep. A significant observation was the upregulation trend in most DE circRNAs, suggesting their potential role in the later stages of fat tail growth in sheep. The study also highlighted that DE circRNAs are linked to specific host genes involved in lipid and fatty acid metabolism. Additionally, binding interactions between DE circRNAs and DE miRNAs were predicted, revealing that certain circRNAs, such as circRNA4175, circRNA1985, and circRNA382, might bind to multiple miRNAs. This indicates that these circRNAs are potential major regulators in the development of the sheep’s fat tail.

### 4.7. Endocrine System

The endocrine system in sheep, a complex network of glands and hormones, plays a crucial role in regulating various physiological processes essential for their growth, reproduction, and overall health [82]. Li et al. (2017) [83] conducted a comprehensive genome-wide analysis of circRNAs within the prenatal and postnatal pituitary glands of sheep. They utilized deep sequencing techniques to detect circRNAs present in the pituitary glands, ultimately identifying a total of 10,226 distinct circRNAs. Among these, 7855 circRNAs exhibited differential expression between the embryonic pituitary gland (PG_E) and the adult pituitary gland (PG_A). These DE circRNAs included 5265 that were upregulated and 2590 that were downregulated. Enrichment analysis of the host genes associated with these DE circRNAs provided insights into their roles in fundamental biological processes within the pituitary gland. Furthermore, a KEGG pathway analysis revealed that circRNAs played a significant role in regulating hormone secretion in the adult pituitary gland. This involvement encompassed pathways such as the thyroid hormone signaling pathway, GnRH signaling pathway, and phosphatidylinositol signaling pathway. In addition to their expression patterns and functional enrichment, the researchers explored the interactions between circRNAs and microRNAs (miRNAs) in sheep. Remarkably, they identified a substantial number of interactions. Of particular interest, they observed that oar_circ_0000059 contained 58 potential binding sites for nine pituitary-related miRNAs, namely miR-103, miR-148, miR-150, miR-16b, miR-181a, miR-19b, miR-23, miR-30, and miR-329. This discovery suggests that oar_circ_0000059 may play a role in the development and endocrine functions of the pituitary gland by acting as a sponge for multiple miRNAs.

**Figure 4:**
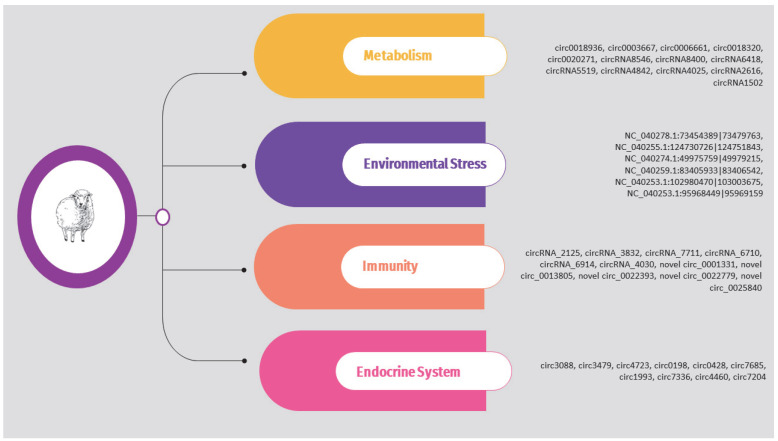
Overview of circRNAs associated with metabolism, environmental stress, immunity, and endocrine system in sheep.

The circRNAs identified in the above studies are also presented in Supplementary Table S2 and Figure 4.

## 5. Circular RNAs in Goat: Shaping Health and Productivity

Goats (*Capra hircus*), with their diverse utility ranging from milk and meat production to fiber, have been a cornerstone in agriculture and the global economy for many years. Their remarkable adaptability to a variety of environments, coupled with efficient reproductive and foraging capabilities, render them invaluable across both developed and developing regions [3]. Notably, over the past 50 years, the global goat population has surged by approximately 240%, a stark contrast to the stable or declining populations of other livestock species [84]. Reflecting their growing prominence, the global production of goat milk has also reached 20 million metric tons, and goat meat production stands at 6.2 million metric tons [85].

Given this rising interest in goats, studies on circRNAs offer critical insights into their health, welfare, and productivity. Thus, this section is dedicated to exploring the latest advancements in circRNA research in goats, underscoring its importance and the potential it holds within the realms of agricultural and genetic research.

### 5.1. Wool

Wool production from goats, especially in the cultivation of specialty fibers like cashmere, plays a vital role in the global textile industry. Cashmere, a keratinized product of secondary hair follicles, is prized for its exceptional softness, lightness, and ability to retain heat, making it a high-value material [86]. The quality and quantity of goat wool depend on a complex combination of genetic factors, diet, and environmental conditions [87,88,89]. Given the significant economic importance of cashmere, there is a growing number of studies focusing on the role of circRNAs in cashmere goats.

In one of the first studies, Zheng et al. (2020) [90] explored the role of circRNAs on cashmere fineness. The research focused on skin samples from two breeds: Liaoning cashmere goats (LCG) and Inner Mongolia cashmere goats (MCG). Using RNA-seq, the team identified 13,320 circRNAs. Among these, 32 were found to be DE between the two breeds, with 17 circRNAs significantly upregulated and 15 downregulated in LCG. These circRNAs originated from 4826 host genes. Subsequently, the researchers conducted GO and KEGG enrichment analyses on the host genes of the DE circRNAs. The top 20 significant GO terms highlighted that keratinization and intermediate filament organization were closely associated with cashmere fiber growth, whereas the most enriched KEGG pathways included the sulfur relay system, sulfur metabolism, and glycosaminoglycan degradation, suggesting their involvement in regulating cashmere fineness. The study also predicted potential circRNA–miRNA interactions for these DE circRNAs. The results indicated a co-expression network comprising 32 DE circRNAs, their host genes, and 244 miRNAs. Notably, circRNA6854 might function as a sponge for several miRNAs. Finally, to identify DE circRNAs in LCG, qPCR was performed on 10 candidate circRNAs from coarse-type skin (CT-LCG) and fine-type skin (FT-LCG) of LCG. The results confirmed that four circRNAs (ciRNA128, circRNA6854, circRNA4154, and circRNA3620) were DE in CT-LCG and FT-LCG, underscoring their potential role in cashmere fineness. Similarly, Hui et al. (2021) [91] aimed to uncover the molecular mechanisms by which ceRNAs regulate the growth and fineness of cashmere by examining the transcriptomes of two types of cashmere goat skin: those from Liaoning cashmere goats (LCG) and Inner Mongolia cashmere goats (MCG). They identified 32 circRNAs that were differentially expressed, of which 17 were upregulated and 15 downregulated in LCG. Subsequently, the researchers constructed a ceRNA network to represent the regulatory interactions among lncRNAs, circRNAs, miRNAs, and mRNAs. They identified circRNA–mRNA interaction pairs that share miRNA binding sites, and the results also indicated significant interaction pairs such as circ6854-miRNA-106-*DSG4* and circ3075-miRNA-129-5p-*TCHH*. Additionally, circ452 and miRNA-2330 were found to act concurrently with *MSX2*, *TCHH*, *KRT35*, and *JUNB*. All the above are potentially key pathways in regulating cashmere fineness.

In a similar approach, Wang et al. (2022) [92] focused on examining the expression patterns and functions of circRNAs in neck skin samples from normal-quality brush hair goats (NHQ group) and superior-quality brush hair goats (HQ group). They successfully identified 61,803 circRNAs, among which 32 showed differential expression between the NHQ and HQ groups, with 13 being upregulated and 19 downregulated. The functional enrichment analysis indicated that the genes from which these DE circRNAs originated were predominantly involved in platelet activation and the focal adhesion signaling pathway. Further analysis of their action mechanisms suggested that these DE circRNAs could also act as sponges for several miRNAs, such as miR-31, miR-125b, miR-let-7a, and miR-149-5p which are known to play significant roles in the growth of goat hair follicle stem cells, as well as in the development and morphogenesis of hair follicles.

In another study, Hu et al. (2023) [93] compared Liaoning cashmere (LC) and Ziwuling black (ZB) goats, focusing on differences in cashmere production and quality. More specifically, skin samples from 6 healthy male goats of each breed were collected during the anagen phase. Of the 11,613 circRNAs detected in caprine skin, 7639 were common to both breeds. Notably, 261 circRNAs were DE between LC and ZB goats, with 115 upregulated and 146 downregulated in LC goats. The most upregulated circRNAs in LC goats included circ_003977, circ_001161, and circ_004733, while the most downregulated were circ_007344, circ_001762, and circ_003528. The study also revealed that the parent genes of these DE circRNAs were largely enriched in GO terms and pathways linked to cashmere fiber traits, such as the canonical Wnt signaling pathway, stem cell growth, epithelial morphogenesis, the MAPK signaling pathway, and cell adhesion molecules pathway. Furthermore, 50 circRNAs were exclusive to ZB goats and 29 to LC goats. The study also examined 8 DE circRNAs as potential miRNA sponges in caprine skin, including 6 upregulated (e.g., circ_003577, circ_007262) and 2 downregulated (e.g., circ_003005) in LC goats. Circ_003577, circ_007262, and circ_001980 were exclusive to LC goats, whereas circ_000353 was unique to ZB goats. These 8 circRNAs collectively had 137 binding sites for miRNAs known to influence hair follicle growth and development. Finally, the study suggests that circRNAs like circ_001980, possibly derived from genes like *CDK19* and *FGFR2*, may regulate differences in cashmere fiber between LC and ZB goats.

Some studies also explored the role of circRNAs in hair follicle development. Shang et al. (2021) [94] explored the expression patterns and functional roles of circRNAs in the development of fetal hair follicles in cashmere goats using high-throughput sequencing. The study involved 12 three-year-old ewes and focused on fetal skin samples collected during four distinct gestational stages (45, 55, 65, and 75 days). They identified a total of 21,784 circRNAs across these stages. Notably, the greatest disparity in circRNA expression was observed between the 65th and 55th days (d65 vs. d55), while the least variation was between the 75th and 65th days (d75 vs. d65). GO and KEGG analyses were conducted on the host genes of the DE circRNAs. The GO analysis highlighted genes involved in biological processes relevant to hair follicle growth and development. Concurrently, the KEGG pathway analysis revealed gene enrichment in several pathways critical for hair follicle development, including the Notch, NF-κB, and PI3K-Akt signaling pathways. Finally, a key discovery of the study was the targeted binding interaction between circRNA3236 and two microRNAs: miR-27b-3p and miR-16b-3p. These interactions were experimentally validated, shedding light on the potential role of circRNA3236 as a ceRNA in the morphogenesis and development of hair follicles. Shang et al. (2022) [95] continued their research, focusing on understanding the role of ceRNA regulatory networks in the development of secondary hair follicles in Inner Mongolia cashmere goats. Similar to their previous study, they collected flank skin samples from fetuses at various pregnancy stages (45, 55, 65, and 75 days) and conducted whole-transcriptional sequencing to analyze circRNA, miRNA, and mRNA expression profiles. Their analysis identified 113 circRNAs associated with the development of secondary hair follicles. Then, they constructed a ceRNA regulatory network comprising 13 circRNAs, 21 miRNAs, and 110 mRNAs associated with the development of secondary hair follicles. Additionally, they confirmed the circular nature of four crucial circRNAs (circRNA2034, circRNA5712, circRNA888, and circRNA9127). A significant discovery was also the identification of circRNA5712 as a pivotal component in the ceRNA network regulating secondary hair follicle development, as they demonstrated the interaction between circRNA5712 and miR-27b-3p and the downstream gene *DLL4*.

The study performed by Shen et al. (2022) also aimed to explore the regulatory networks and expression profiles of specific circRNAs in the secondary hair follicles (SHFs) of cashmere goats across different SHF cycles, including the growth (anagen), regression (catagen), and resting (telogen) phases [96]. The research involved analyzing skin tissues from cashmere goats. Using qRT-PCR, the researchers observed a significant upregulation in the expression of nine circRNAs during the anagen phase as compared to the telogen phase. These circRNAs included circRNA-BOC, circRNA-CAMSAP1, circRNA-VWA8, circRNA-ERCC6, circRNA-PLIN2, circRNA-TIAM1, circRNA-TXNRD3, circRNA-TENT2, and circRNA-TENT4A. Furthermore, they constructed circRNA–miRNA–mRNA networks for these nine circRNAs, and they discovered that they were intricately linked to various signaling pathways, such as the Wnt, TGF-beta, mTOR, MAPK, and axon guidance pathways.

Similarly, Gao et al. (2023) [97] conducted a study on the ceRNA regulatory network influenced by circRNAs in the skin tissues of cashmere goats during fetal stages (75 and 125 days). They identified 394 DE circRNAs in the E75 and E125 groups, with 154 being upregulated and 240 downregulated. This notable variation in circRNA expression during these stages points to their potential role in the development of secondary hair follicles (SHF). Additionally, the researchers constructed ceRNA networks, placing particular emphasis on miR-184, which, according to their study, promotes cell proliferation, inhibits apoptosis, and affects the cell cycle by competitively liberating *FGF10* (Fibroblast Growth Factor 10). Finally, within this network, they found that circRNA-0001141 competitively binds to miR-184.

In a different study, Hui et al. (2022) [98] investigated *N*^6^-methyladenosine (m^6^A) modifications in circRNAs within cashmere goat skin tissue, particularly during the anagen stage of hair follicle growth. m^6^A is known as the most abundant modification in linear RNA molecules, and recent findings have revealed its presence in circRNAs as well, with specific temporal and spatial expression patterns. The team identified 15 m^6^A-circRNAs in the skin tissue, six of which exhibited higher expression in the anagen phase than in the telogen phase. These six m^6^A-circRNAs were implicated in complex miRNA-mediated regulatory pathways. The study highlighted the enrichment of key signaling pathways, including TGF-beta and stem cell pluripotency, which are critical in hair follicle physiology, suggesting a significant role of these m^6^A-circRNAs in the development of secondary hair follicles (SHF) and cashmere growth. Notably, four of the identified m^6^A-circRNAs, such as m^6^A-circRNA-ZNF638 and -CAT, followed expression patterns similar to those of their host genes in SHFs. However, two m^6^A-circRNAs, m^6^A-circRNA-STAM2 and -CAAP1, exhibited expression patterns inconsistent with their host linear RNAs, possibly due to their regulation by other mechanisms.

### 5.2. Milk Production

The dairy goat industry has seen significant growth in recent years, with the global goat population increasing notably over the past decade, surpassing the growth rates of sheep and cattle [99,100]. A key contributor to this success is the extensive breed diversity, encompassing over 500 breeds, combined with goats’ remarkable ability to adapt to various environmental conditions. Furthermore, goat milk is attracting attention in developed countries for its sensory qualities and lower allergenic potential compared to cow milk [100].

Thus, the following section will delve into the critical role of circRNAs in mammary gland health and milk production. Understanding these molecular mechanisms is crucial for optimizing dairy goat milk yield and improving the health of the mammary gland, aspects central to advancing the dairy goat industry.

In one of the first studies, Ma et al. (2019) [101] focused on understanding the genetic aspects of goat lactation. They employed RNA-seq on mammary tissue samples collected at two key lactation stages: early lactation (L-5 d) and mature lactation (L-30 d). Their analysis identified a total of 37,818 circRNAs, among which 864 exhibited differential expression when comparing mature to early lactation stages (611 upregulated and 253 downregulated). The researchers also constructed ceRNA networks to explore the roles of circRNAs in lactation. To predict the functions of these ceRNAs, GO and KEGG enrichment analyses were conducted on the coding genes interacting with circRNAs. The GO analysis revealed significant involvement in processes such as the positive regulation of gene silencing by miRNA, regulation of mRNA stability, and positive regulation of striated muscle tissue development. Meanwhile, the KEGG pathway analysis highlighted pathways critical to lactation, including ECM-receptor interaction, PI3K-AKT signaling pathway, ABC transporters, and pathways related to fat and protein digestion and absorption. Overall, these findings suggest a substantial role of circRNAs in regulating lactation processes in goats.

In a different study, Chen et al. (2022) [102] explored the role of circRNAs in fatty acid metabolism within the mammary glands of dairy goats. Using RNA-seq, they identified 211 DE circRNAs during early and peak lactation phases. Among these, 116 circRNAs were upregulated, and 105 were downregulated in peak lactation compared to early lactation. The researchers also conducted a KEGG analysis on the host genes of these DE circRNAs, uncovering significant enrichment in several metabolic and biosynthetic pathways, including the mRNA surveillance pathway, nicotinate and nicotinamide metabolism, pyrimidine and purine metabolism, lysine degradation, mismatch repair, and mannose type O-glycan biosynthesis. They notably focused on circ007071, which was found to be 12.02 times more expressed during peak lactation compared to early lactation. This circRNA enhances the production of triglycerides, cholesterol, and certain saturated fatty acids. Furthermore, they showed that overexpressing circ007071 reduces miR-103-5p levels, leading to decreased triglyceride synthesis and increased peroxisome proliferator-activated receptor γ (*PPAR*γ), a gene targeted by miR-103-5p. This indicates that circ007071 regulates milk fat synthesis by counteracting miR-103-5p’s inhibition of *PPAR*γ, promoting the synthesis of TAG and saturated fatty acids.

In a very similar approach, Jiao et al. (2022) [103] conducted a study to explore the influence of circRNAs, particularly circ003429, on unsaturated fatty acid synthesis in goat mammary gland epithelial cells (GMECs). Initially, the team identified 215 DE circRNAs between the early lactation and dry/nonpregnant stages in goat mammary gland tissues, suggesting a role for circRNAs in fatty acid synthesis. The KEGG enrichment analysis revealed that the host genes of these circRNAs were involved in various pathways, including cancer, metabolic pathways, PI3K-Akt signaling, actin cytoskeleton regulation, and apoptosis. The study then focused on circ003429, which was significantly upregulated in GMECs. It was discovered that circ003429 not only inhibits the synthesis of triglycerides (TAG) but also reduces the levels of unsaturated fatty acids. Additionally, the knockdown of circ003429 resulted in decreased expression of genes associated with unsaturated fatty acid synthesis and a subsequent reduction in unsaturated fatty acid content in GMECs. Further investigation showed that circ003429 acts as a sponge for miR-199a-3p, therefore affecting the expression of its target gene, *YAP1*. This mechanism suggests that circ003429 plays a crucial role in the regulation of unsaturated fatty acid synthesis in the dairy goat mammary gland.

Furthermore, Xuan et al. (2023) [104] aimed to explore the expression patterns of circRNAs in non-lactating goat mammary gland tissue, identifying those associated with mammary gland development and lactation regulation. They analyzed mammary gland tissue from three stages: late lactation (LL), dry period (DP), and late gestation (LG). RNA-seq identified 11,756 circRNAs, with the highest abundance exclusively expressed during the DP and the lowest in the LL stage. GO analysis of circRNA host genes showed enrichment in functions like histone modification and cell polarity, while KEGG pathway analysis revealed the enrichment of endocytosis and ErbB signaling pathways. Differential expression analysis highlighted circRNAs associated with mammary gland development and lipid storage regulation in the LL vs. DP comparison. In contrast, the DP vs. LG comparison revealed the downregulation of lactation-related genes and the upregulation of wound-healing genes. The LL vs. LG comparison also showed enrichment in response to insulin. A total of 218 circRNAs were DE across the three groups, with the most significant changes observed in the DP vs. LG group. The study noted stage-specific circRNA expression patterns during mammary gland tissue involution and remodeling, highlighting the dynamic nature of circRNA expression. Furthermore, the study constructed circRNA–miRNA–mRNA competitive endogenous RNA (ceRNA) networks related to mammary gland development, immunity, substance metabolism, and cell apoptosis. These networks suggest that circRNAs may influence various physiological processes, including apoptosis, growth, metabolism, tissue remodeling, intercellular interactions, disease infection, and immune response, by competitively binding with miRNAs.

**Figure 5:**
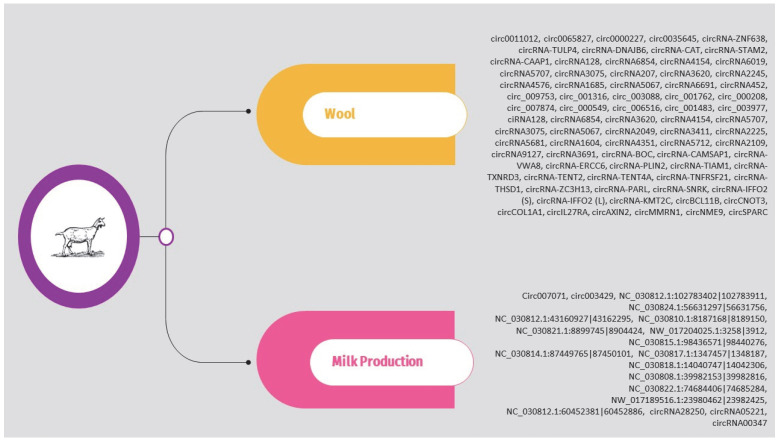
Overview of circRNAs associated with wool and milk production in goat.

The circRNAs identified in the above studies are presented in Supplementary Table S3 and Figure 5.

### 5.3. Muscle Growth and Development–Meat Quality

Goat meat presents a promising, sustainable alternative in the realm of red meats. Characterized by its low environmental impact, goat farming typically requires less resource-intensive inputs compared to other livestock and is well-suited for free-range practices. Moreover, goat meat is recognized for its health benefits, particularly its lean profile with lower fat content, making it an appealing choice for health-conscious consumers. Despite these advantages, goat meat’s full potential in the meat trade market remains under-realized [105]. This gap is also mirrored in the limited but growing body of research on the roles of circRNAs in goat muscle development, as presented in this section.

In an interesting study, Ling et al. (2020) [106] examined the role of circRNAs in the development of skeletal muscle in Anhui White Goats (AWG), covering seven developmental stages from fetal to postnatal growth. The researchers collected *longissimus dorsi* muscle samples at various gestational and postnatal stages and constructed 21 RNA sequencing libraries to analyze the circRNA profiles. This study identified a total of 9090 circRNAs, underscoring the complexity and diversity of their formation, as a single host gene can produce multiple circRNA isoforms with distinct expression profiles. Of these circRNAs, 2881 were differentially expressed. Furthermore, the researchers identified 1118 DE circRNAs under stringent conditions, which displayed four main expression trends. An interesting finding was that chromosome 1 harbored the highest number of DE circRNAs. This suggests a significant contribution of chromosome 1 to circRNA production during muscle growth. Finally, the study also outlined three transitional stages in skeletal muscle development: Stage 1 (F45 to F90), associated with muscle satellite cell proliferation and muscle fiber structure; Stage 2 (F90 to B1), marking the initiation of cytoplasmic attachment to the actin cytoskeleton; and Stage 3, involving the cGMP-PKG signaling pathway.

Similarly, Zhou et al. (2022) [107] aimed to identify and analyze the expression profiles of circRNAs in the *longissimus dorsi* muscle of Wu’an goats at different ages, focusing on their potential role in muscle development. More specifically, muscle tissue from 1-month-old and 9-month-old Wu’an goats was used. High-throughput whole-transcriptome sequencing revealed 686 DE circRNAs. GO analysis linked the host genes of these DE circRNAs to various cellular processes, while KEGG pathway analysis highlighted pathways crucial for muscle growth, including the oxytocin signaling pathway, the FoxO signaling pathway, and the GMP-PKG signaling pathway. The study also established a circRNA–miRNA–mRNA network involving 201 circRNAs, 85 miRNAs, and 581 mRNAs. Furthermore, luciferase reporter assays validated the binding sites between circRNAs and target miRNAs, identifying several circRNAs like novel_circ_0005319, novel_circ_0005934, and novel_circ_0000134 potentially influential in skeletal muscle growth and development. Fan et al. (2023) also utilized high-throughput sequencing and bioinformatics to examine circRNA expression in the *longissimus dorsi* muscle of goats at two developmental stages: D75 fetus and D1 kid [108]. They identified 831 DE circRNAs and analyzed their host genes, uncovering significant associations with biological processes like calcium-dependent protein kinase activity and muscle alpha-actinin binding, and pathways including adherens junction, ubiquitin-mediated proteolysis, and the FoxO signaling pathway. The study then focused on circUBE3A, which was highly expressed in kid skeletal muscle and differentiated myoblasts. They discovered that suppressing circUBE3A enhances cell proliferation and differentiation, and it interacts with miR-28-5p to promote *HADHB* expression, therefore aiding in myoblast proliferation and differentiation.

In a different approach, Shet et al. (2022) [109] conducted a study to identify and characterize DE circRNAs in the *longissimus dorsi* muscle of two goat breeds with contrasting traits in muscle growth and fatness. They analyzed muscle samples from 5 Liaoning cashmere (LC) goats and 5 Ziwuling black (ZB) goats, and RNA-seq revealed the identification of 8781 circRNAs in LC goats and 8872 circRNAs in ZB goats. Of these, 6778 circRNAs were common to both breeds. A notable finding was the high expression of circ_001086, derived from the LIM domain 7 (*LMO7*) gene, in the muscle tissues of both LC and ZB goats. Furthermore, out of the 10,875 circRNAs identified in the caprine *longissimus dorsi* muscle, 214 were DE between the two breeds. In LC goats, 85 circRNAs were upregulated, and 129 were downregulated compared to ZB goats. The most upregulated circRNA in LC goats was circ_008092, while the most downregulated was circ_003628, derived from the *MYH4* gene. The study also annotated 195 parent genes for these 214 DE circRNAs. The most significant GO terms identified were related to connective tissue development and syncytium formation. Additionally, several GO terms relevant to skeletal muscle hypertrophy were identified. Regarding KEGG pathways, the most enriched were pathways associated with skeletal muscle growth and intramuscular fat deposition, such as the Rap1 signaling pathway, cGMP-PKG signaling pathway, cAMP signaling pathway, etc. Finally, the researchers constructed circRNA–miRNA–mRNA networks, demonstrating that circRNAs act as sponges for miRNAs involved in muscle development and intramuscular fat deposition.

### 5.4. Immunity

Although the immune system is fundamental to an animal’s overall health and productivity, research into the role of circRNAs in the immunity of goats remains surprisingly sparse, with just a handful of studies addressing this critical area.

In the first study, Chen et al. (2021) [110] explored how goats’ immune system responds to infection by *Pasteurella multocida*, a bacterial pathogen. They conducted transcriptome sequencing on lung tissues from goats exposed to *P. multocida* and analyzed the expression profiles of circRNAs, messenger RNAs (mRNAs), and miRNAs. Their findings showed significant alterations in transcriptome expression in the lung tissues of infected goats. Specifically, 138 circRNAs exhibited significant differential expression compared to the control group, with 89 upregulated and 49 downregulated. GO analysis of these circRNAs’ source genes highlighted their involvement in immune-related biological processes such as inflammatory response, immune response, and cytokine-mediated signaling. Additionally, the study identified enrichment in several KEGG pathways, including those related to sugar metabolism and glycerophospholipid metabolism. Notably, the researchers constructed ceRNA networks and identified circ_04689 as a key circRNA, which influenced the downregulation of 13 target miRNA transcripts, underscoring its significant role in the immune response to *P. multocida*.

In the second study, Wang et al. (2023) [111] explored the role of circRNAs in the immune functions of goat submandibular glands (SMG) across different ages. More specifically, transcriptome sequencing of SMG tissues from 1, 12, and 24-month-old goats revealed 1699 DE circRNAs. Interestingly, the study found a notable decline in the expression of key circRNAs, lncRNAs, and immune-related genes with increasing age. This suggests a correlation between noncoding RNA levels and the diminishing immune function of the salivary glands in aging goats. Additionally, the study emphasized that through ceRNA network and functional enrichment analysis, they identified that 13 circRNAs and 14 lncRNAs are potentially regulating five crucial immune genes: *ITGB2*, *LCP2*, *PTPRC*, *SYK*, and *ZAP70*. This regulation occurs via competitive interactions with miRNAs, such as miR-141-x, miR-29-y, and chi-miR-29b-3p. These interactions are significant for pathways like natural killer cell cytotoxicity and T cell receptor signaling, among other immune-related processes.

### 5.5. Metabolism

Regarding metabolism and the role of circRNAs, only one study was identified. More specifically, Zhong et al. (2022) [112] conducted a study on the expression of circRNAs in the rumen of goats during their fetal (days 60 and 135 of gestation, F60 and F135) and prepubertal periods (days 60 and 150 of age, BW30 and AW150). They identified 1518 DE circRNAs across these four developmental stages. Specifically, 76 DE circRNAs were found between F60 and F135, 798 between F135 and BW30, and 125 between BW30 and AW150. These results indicate distinct circRNA expression profiles in fetal and prepubertal stages. Furthermore, GO and KEGG pathway analyses showed that the host genes of the DE circRNAs were primarily involved in cell proliferation, division, and apoptosis pathways. Finally, the researchers constructed circRNA–miRNA networks, and interestingly, circ5813-1, which is predicted to interact with 12 miRNAs, emerged as a key player in rumen development. The networks constructed also showed the involvement of circRNAs in cell proliferation and apoptosis signaling pathways.

**Figure 6:**
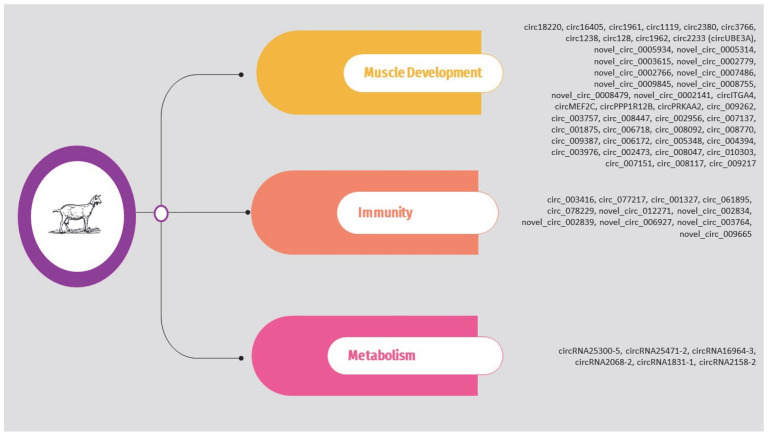
Overview of circRNAs associated with muscle development, immunity, and metabolism in goat.

As previously, the circRNAs identified in the above studies are presented in Supplementary Table S3 and Figure 6.

## 6. Discussion

Improving animal health and welfare is crucial for the livestock industry, not only for enhancing the quality of food products but also for positively influencing consumer perception and acceptance. Advances in molecular biology and genetics, particularly through cutting-edge techniques like high-throughput sequencing, are critical in achieving this goal. Increasingly, studies underscore the significance of ncRNAs in regulating various cellular processes, also highlighting their potential as biomarkers. In this context, research on circRNAs stands out as exceptionally promising as these studies provide new insights into the molecular dynamics that govern animal health and welfare. By uncovering the roles of circRNAs in various physiological processes, such research could lead to the development of innovative biomarkers for the early detection of diseases, evaluation of stress responses, and overall monitoring of livestock well-being. This not only aligns with consumer concerns about animal welfare but also aids in producing higher-quality products. This combination of addressing consumer expectations and ensuring product quality makes circRNA research scientifically significant and economically valuable in the livestock industry. Therefore, here, we have reviewed the role of circRNAs in livestock, with a special emphasis on health, welfare, and productive characteristics.

Especially regarding the applicability of the findings presented in this review, the unique closed-loop structure of circRNAs enhances their cellular stability, positioning them as promising biomarkers for disease diagnosis and animal health monitoring. Their application in agriculture could transform farm management, improve animal welfare, ensure food safety, and promote environmental sustainability, benefiting both producers and consumers. This shift towards circRNA-based monitoring represents a significant advancement in agricultural practices.

For farmers, the use of circRNAs as biomarkers heralds a new era of precision agriculture. The benefits of this approach span various aspects of farm management, from disease prevention to enhancing animal welfare, ultimately resulting in increased productivity and economic gains. First, circRNAs, due to their high stability and specificity, can facilitate the early detection of diseases in livestock. This early diagnosis capability allows for timely interventions, preventing the spread of infectious diseases within the herd and reducing the overall incidence of illness. A relevant example can be found in Figure 6, which presents specific circRNAs that indicate *Pasteurella multocida* infections in goats, as highlighted in the study by Chen et al. (2021) [110]. This review also discusses the potential of certain circRNAs in disease detection, which not only impact animal health but also influence productivity. For example, circRNAs shown in Figure 2 have been identified as potential biomarkers for *Staphylococcus aureus*-induced mastitis in cattle, a condition that significantly affects milk production, as reported by Bai et al. (2022) [39]. Additionally, monitoring stress indicators, such as circRNAs associated with heat stress in cattle, as shown in Figure 2, and assessing animals’ nutritional status and metabolism through circRNA levels enables farmers to optimize living conditions and diets. This ensures that the animals are not only healthier but also more productive. This tailored approach, especially regarding nutrition, can lead to more efficient feed use, improved growth rates, and overall better health for the animals. Moreover, circRNAs offer a promising avenue for genetic selection to enhance desirable traits, such as meat quality. By identifying circRNAs associated with favorable meat characteristics, breeders and farmers can improve their breeding strategies to select animals that are more likely to produce premium meat. This circRNA-informed genetic selection could lead to livestock that are healthier and genetically predisposed to produce meat with superior taste, texture, and nutritional content. Importantly, circRNAs linked to muscle growth and meat quality have been identified in all species examined in the present review, as depicted in Figure 1, Figure 3 and Figure 6. Similarly, circRNAs that impact lactation, mammary gland development, and the metabolic processes related to milk production (Figure 1, Figure 3 and Figure 5) can help in selecting animals, potentially enhancing milk production and quality. This approach can be applied to other desirable traits, like wool production (Figure 3) and even disease resistance, as seen in circRNAs associated with resilience against *Escherichia coli* F17-induced diarrhea, as reported by Jin et al. (2018) [77] (Figure 4). The cumulative effect of early disease detection, improved animal welfare, enhanced reproductive management, and optimized nutrition strategies leads to considerable economic benefits for farmers, as healthier animals have higher productivity in terms of milk yield, growth rates, etc. Furthermore, reducing disease incidence and improving animal welfare can also enhance the farm’s sustainability and reputation, potentially leading to better market opportunities.

It should also be noted that implementing circRNA biomarkers in animal health management has profound ripple effects on consumers. Enhanced disease management and animal welfare practices lead to the production of safer, higher-quality meat, dairy, and other products. The strategic use of circRNAs ensures livestock are raised in stress-minimized environments, resulting not only in safer but potentially more nutritious food products. This meets the increasing consumer demand for transparency regarding animal welfare in the food production chain. Using circRNAs to ensure the health and well-being of livestock can assure consumers that the products they purchase come from well-cared-for animals. This can build consumer trust in brands and products and align with the growing demand for ethically produced food. Additionally, by enabling early disease detection and management, circRNA biomarkers can decrease the need for antibiotics in animal farming. This is essential for addressing the global issue of antibiotic resistance, which is a significant concern for human health. Consumers benefit from knowing that their food comes from systems that use antibiotics responsibly, reducing the risk of antibiotic-resistant bacteria entering the food chain. Lastly, incorporating advanced technologies like circRNA biomarker monitoring in the food production process can enhance the transparency and traceability of food products. Consumers receive information about the health and welfare conditions under which their food was produced, enabling them to make more informed purchasing decisions.

As with all reviews, ours also encompasses certain limitations. First, we confined our selection to in vivo studies, prioritizing them for their potential to provide a comprehensive list of circRNAs as biomarkers for animal welfare, health, and other related areas. In vivo studies, being conducted within living organisms, offer profound insights into physiological and biochemical processes in a complex, integrated environment and are typically more applicable to real-world scenarios. However, it is possible that with this approach, some information has been missed, particularly concerning the interactions between circRNAs, miRNAs, and mRNAs, which are often explored in cell line experiments. Additionally, our review specifically focuses on studies related to animal welfare, health, and productive traits while deliberately excluding those centered on reproduction. This deliberate focus was chosen to enable a more thorough exploration of circRNAs in contexts that are vitally significant for animal management, welfare policies, and productivity strategies. While reproductive studies are undoubtedly crucial in animal science, they represent a separate research area with distinct complexities and outcomes. Lastly, by restricting our review to articles published in English, we acknowledge the possibility of overlooking pertinent research published in other languages, which could limit the scope of our findings.

This review is also subject to several limitations arising from the nature of the included studies. First, a notable concern is the small sample sizes in some studies, which could potentially lead to less reliable results. Additionally, there is considerable variability in how differential expression of circRNAs is defined across these studies. The use of diverse fold change thresholds for determining differential expression may introduce discrepancies and hinder direct comparison and interpretation of the results. Another critical limitation is the lack of information regarding the mapping and circRNA calling processes used in most of the studies. Mapping and circRNA calling are crucial steps in circRNA research. The specific methods and algorithms used for these processes can significantly affect the identification and quantification of circRNAs. Unfortunately, the lack of detailed methodological descriptions in some studies makes it difficult to evaluate the exact mapping and circRNA calling techniques employed. This absence of information could introduce variation in the detection and quantification of circRNAs. Such variations, in turn, may impact the comparability and overall interpretation of the findings. It is important to note that inconsistent naming of circRNAs is a major concern in the scientific community, as it affects clarity and reproducibility. Recent efforts aim to standardize this nomenclature. Specifically, Chen et al. (2023) [113] discuss the rapid increase in circRNA identification and emphasize the need for a unified naming approach.

Our study, despite its limitations, also possesses significant strengths. First, it stands out as a comprehensive review exploring the role of circRNAs in major livestock species, namely cattle, goats, and sheep. This focus on key species provides valuable insights relevant to a broad spectrum of animal health and agricultural research. Additionally, we adopted a rigorous selection criterion, excluding studies with inadequate methodology or lacking essential information. This approach ensures that our review is grounded in robust and reliable data, contributing to its credibility and value. Moreover, our review is characterized by its inclusiveness, which was achieved through an extensive search across two leading databases: PubMed and Web of Science. This expansive search strategy has allowed us to capture a wide range of relevant studies, enhancing the comprehensiveness of our review.

Regarding future directions, research on circRNAs in livestock remains in its early stages, with fewer than 20 studies identified for each animal species, highlighting a broad knowledge gap. Despite goats and sheep being slightly more studied (*n* = 20 and *n* = 19, respectively), all species, including cattle (*n* = 12), are significantly understudied. This underscores the need for more comprehensive research across all livestock to better understand circRNA roles in animal health and welfare, facilitating the development of biomarkers. Furthermore, there is an uneven focus on specific traits within each species. For instance, in cattle, research predominantly centers on milk production (*n* = 6) and heat stress (*n* = 4), with fewer studies on muscle development and meat quality (*n* = 1), and immunity (*n* = 1). In sheep, studies are more varied, covering muscle growth (*n* = 6), milk production (*n* = 4), wool quality (*n* = 3), metabolism (*n* = 2), and immunity (*n* = 2). However, limited research is observed on environmental stressors (*n* = 1) and the endocrine system (*n* = 1). Similarly, goat research primarily examines wool (*n* = 9), milk production (*n* = 4), and muscle growth (*n* = 4), with fewer studies on immunity (*n* = 2) and metabolism (*n* = 1). Thus, future research should aim to address these gaps, especially in less-studied areas and species. Additionally, most of the circRNA–miRNA interactions identified in the studies presented here are based on bioinformatics predictions. Therefore, experimental validation of these interactions and the exploration of circRNA networks are essential for advancing our understanding. Finally, despite the potential of circRNAs as biomarkers for monitoring animal health and welfare, significant challenges remain. The lack of a standardized protocol for circRNA quantification impedes consistent comparison of expression profiles across different laboratories. Thus, the development of standardized methods to minimize biases and normalize raw data is crucial. To establish the efficacy of circRNAs as biomarkers, it is imperative to undertake large-scale studies. These studies compare circRNA expressions in various livestock species under diverse stress conditions, varying in intensity and duration, to validate their applicability.

## 7. Conclusions

Improving animal health and welfare plays a pivotal role in meeting consumer expectations and ensuring product quality in the livestock industry. This importance is underscored by national and international policies that advocate for an equitable, sustainable, and resilient food system capable of feeding the global population and grounded in the fundamental principle of respecting animal welfare. Consequently, there is a pressing need for innovative and measurable biomarkers that can offer a precise evaluation of an animal’s well-being and quality of life, a requirement that traditional methods often fail to meet.

This review explores the potential of circRNAs in elevating livestock health and welfare standards, with a specific focus on their influence on productive characteristics, resilience to environmental stresses, such as heat stress, and disease control. CircRNAs are emerging as promising biomarkers due to their stability and detectability in various bodily fluids, making them well-suited for non-invasive sampling methods like blood, saliva, milk, etc. This unique feature streamlines the diagnostic process minimizes stress for the animals, and enables easier health monitoring and early disease identification.

However, despite the promising aspects of circRNA, the field is currently navigating through significant challenges, such as the absence of standardized protocols for circRNA quantification and a unified nomenclature system. As a result, the emerging field of circRNA research holds tremendous promise for revolutionizing animal welfare and management strategies, but overcoming these hurdles is essential for circRNAs to fulfill their potential as key biomarkers in stress and health management within livestock.

## Data Availability

Not applicable.

## Supplementary Materials

The following supporting information can be downloaded at https://www.mdpi.com/article/10.3390/ani14050733/s1, Table S1: Overview of study characteristics regarding the role of circRNAs in cattle; Table S2: Overview of study characteristics regarding the role of circRNAs in sheep; Table S3: Overview of study characteristics regarding the role of circRNAs in goat.
